# Placental surface area mediates the association between *FGFR2* methylation in placenta and full-term low birth weight in girls

**DOI:** 10.1186/s13148-018-0472-5

**Published:** 2018-03-22

**Authors:** Fu-Ying Tian, Xi-Meng Wang, Chuanbo Xie, Bo Zhao, Zhongzheng Niu, Lijun Fan, Marie-France Hivert, Wei-Qing Chen

**Affiliations:** 1 Department of Medical Statistics and Epidemiology, Guangzhou Key Laboratory of Environmental Pollution and Health Assessment, Guangdong Provincial Key Laboratory of Food, Nutrition and Health, School of Public Health, Sun Yat-sen University, Room 715, 74 Zhongshan Road 2, Guangzhou, 510080 Guangdong China; 2 Department of Cancer Prevention Research, State Key Laboratory of Oncology in South China, Collaborative Innovation Center for Cancer Medicine, Sun Yat-sen University Cancer Center, Guangzhou, China; 3 Children’s Hospital Boston and Department of Biological Chemistry and Molecular Pharmacology, Harvard Medical School, Boston, MA 02115 USA; 4 Department of Epidemiology and Environmental Health, School of Public Health and Health Professions, State University of New York at Buffalo, 265 Farber Hall, Buffalo, NY 14214 USA; 5 Department of Population Medicine, Harvard Medical School, Harvard Pilgrim Health Care Institute, 401 Park Drive, Suite 401, Boston, MA USA; 6 Diabetes Center, Massachusetts General Hospital, 50 Staniford Street, Boston, MA USA; 7 Department of Medicine, Université de Sherbrooke, 3001 12th Avenue North, Sherbrooke, Québec Canada; 8 Centre de recherche du Centre Hospitalier Universitaire de Sherbrooke, 3001 12th Avenue North, wing 9, door 6, Sherbrooke, Québec Canada; 9 Department of Information Management, Xinhua College, Sun Yat-sen University, Guangzhou, Guangdong China

**Keywords:** *FGFR2*, DNA methylation, Placental surface area, Low birth weight, Mediation effect

## Abstract

**Background:**

Fibroblast growth factor receptor 2 (*FGFR2*) gene encodes a protein of the fibroblast growth factor receptor family. *FGFR2* gene expression is associated with the regulation of implantation process of placenta which plays a vital role in fetal growth. DNA methylation is widely known as a mechanism of fetal growth. However, it is unclear whether and how DNA methylation of *FGFR2* gene in the placenta is associated with full-term low birth weight. This case-control study aims to explore the links between *FGFR2* methylation in placenta and full-term low birth weight and to further examine the mediation effect of placental surface area on this association.

**Results:**

We conducted analyses for each of the five valid CpG sites at *FGFR2* in 165 mother-baby pairs (86 FT-LBW vs. 79 FT-NBW) and found that per one standard deviation increase in the DNA methylation of CpG 11 at *FGFR2* was associated with 1.64-fold higher risk of full-term low birth weight (OR = 1.64, 95% CI = [1.07, 2.52]) and 0.18 standard deviation decrease in placental surface area (*β* = − 0.18; standard error = 0.08, *p* = 0.02). The mediation effect of placental surface area on the association between DNA methylation and full-term low birth weight was significant in girls (OR = 1.38, 95% CI = [1.05, 1.80]) but not in boys. The estimated mediation proportion was 48.38%.

**Conclusion:**

Our findings suggested that placental surface area mediated the association between DNA methylation of *FGFR2* in placenta and full-term low birth weight in a sex-specific manner. Our study supported the importance of placental epigenetic changes in placental development and fetal growth.

**Electronic supplementary material:**

The online version of this article (10.1186/s13148-018-0472-5) contains supplementary material, which is available to authorized users.

## Background

Low birth weight (LBW), defined as the newborn birth weight less than 2500 gram (g) [1], is one of the most common adverse birth outcomes that affects about 7.2% of pregnancies in China [2] and as much as 15–20% worldwide [3]. LBW not only increases morbidity and mortality in infants but is also associated with increased susceptibility to chronic non-communicable diseases in adulthood, such as type 2 diabetes, obesity, hypertension, stroke, and cardiovascular diseases [4–10]. LBW is the result of either preterm birth or intrauterine growth retardation (IUGR), or both [11]. To distinguish the IUGR-driven LBW from prematurity LBW, in this study, we only focused on full-term low birth weight (FT-LBW), the LBW with gestational age from 37 to 42 weeks [12].

The placenta is a temporary organ that supports fetal growth and development [13]. LBW can be caused by placental development insufficiency [14] which manifests in different ways including lower placental weight [15–17], shorter breadth of the placental surface [18], and less placental surface area. Placental surface area reflects the invasion of trophoblasts into the maternal decidua and the interface development [19], which affects mother-fetal substance transmission and fetal growth [20]. However, the molecular mechanism of placental development is still poorly understood.

Fibroblast growth factors (FGFs) are critical in the regulation of implantation process of the placenta, including trophoblast differentiation, hormone production [21], and migration [22]. Additionally, upregulated FGF2 signaling pathways participate in the regulation of human placental artery endothelial cell proliferation and angiogenesis [23–27]. FGFs carry out these regulation functions by binding to their receptors (FGFRs), and one of these receptors is the fibroblast growth factor receptor 2 (FGFR2), a high-affinity receptor of most FGFs [18]. Animals and human studies have demonstrated that FGFR2 expresses on the membrane of trophoblast [28–30]. Furthermore, inhibited *FGFR2* expression causes decrease in trophoblast formation and delayed the timing of trophoblast outgrowth [31, 32]. Therefore, it is worth looking more closely into the associations of *FGFR2* with placental development and fetal growth.

DNA methylation is a well-documented mechanism of fetal growth [33]. The importance of DNA methylation lies in the fact that DNA methylation participates in regulating gene expression [34]; also, DNA methylation was proved to be a critical component of fetal programming and long-term onset diseases [35]. LBW has been found to be associated with placental DNA methylation on a general level or of target genes (hypomethylated *IGF2* [36], *HUS1B* [37], and hypermethylated *HSD11B2*, *WNT2*, and *AHRR* [38, 39]). Studies have also suggested the vital role of DNA methylation in placental development [40–42]; however, the associations between DNA methylation of specific genes and placental development remain largely unknown. Hence, it is valuable to determine the association between DNA methylation of placenta itself and development of the placenta, and fetal growth, also.

We hypothesized that *FGFR2* DNA methylation changes might contribute to maldevelopment and poor implantation of the placenta, followed by fetal growth disruption. Here, this study aims to investigate the association of placental *FGFR2* methylation with placental development and FT-LBW and to investigate whether placenta mediates the association between placental *FGFR2* methylation and FT-LBW.

## Methods

### Study design and subjects

A case-control study design was employed. FT-LBW (< 2500 g at birth) mother-baby pairs were defined as cases, while those with birth weight in the range of 2500 to 4000 g and with a gestational age of 37 to 42 weeks (full-term normal birth weight, FT-NBW) were regarded as controls. During September 2009 and March 2011, 86 FT-LBW and 79 FT-NBW mother-baby pairs were enrolled at the maternity and child health care hospitals of two cities (Foshan and Shenzhen) in China. The cases and controls were individually matched for gestational age (± 7 days), pre-pregnant BMI (± 1.5), parity (the same), and newborn gender (the same). We excluded the subjects if (a) the mothers had heart disease, hepatitis, kidney disease, hypertension (gestational hypertension), diabetes mellitus (gestational diabetes mellitus), hyperthyreosis, anemia, or tuberculosis; (b) the mothers used hazardous drugs to the fetus; (c) the mothers had alcohol abuse; (d) the mothers had multiple gestation; (e) the newborns had hereditary disease or congenital malformation.

### Data collection

We surveyed the pregnant women face-to-face using questionnaires to inquire about their demographic characteristics, alcohol and tobacco use, environmental tobacco smoke (ETS) exposure during pregnancy, medical history, and reproductive history. We collected information of last menstrual, pre-pregnancy body mass index (BMI), parity, placental weight and size, birth weight, birth length, head circumference (HC), and newborn gender from the medical records of mothers and newborns. We estimated gestational age at delivery by subtracting the last menstrual period (LMP) date from the delivery date or by the ultrasound if the ultrasound estimation differed from the LMP estimation by more than 10 days.

### Measurements

Newborn birth weight was measured using SECA baby weight balance in a scale interval of 5 g, and birth length and HC were measured by tape in a scale interval of 1 mm. The placental surface of the mother side was recognized as an ellipse. The maximal diameter of the surface and its perpendicular bisecting diameter were considered as major axis and minor axis. The placental surface area was calculated using the formula for the area of the ellipse: major axis × minor axis × *π*/4.

### Tissue sample collection

Placental tissues were collected immediately after delivery. Approximately 1 cm^3^ in size samples were cut from the placental maternal side without the surficial membrane, in the middle of the line between the umbilical cord insertion point and the edge of the placenta, avoiding calcification points. The tissue samples were washed in cold physiological saline until it became pale, then cut into small pieces and stored in a − 80 °C freezer.

### DNA extraction

DNAs from 165 maternal side placental tissue samples were isolated using TIANamp Genomic DNA Kit (TIANGEN Biotech, Beijing, China). DNA quality was assessed by Thermo Scientific™ NanoDrop 2000 (Thermo Scientific™, San Jose) and DNA gel electrophoresis (Major science). The DNA molecular size of all samples were more than 10 Kb, OD260/280 ≥ 1.8**,** OD260/230 ≥ 1.5, and the nucleic acid concentration of all samples was in 0.2–2.0 μg/μL.

### DNA methylation measurement using Sequenom MassARRAY EpiTYPER

We measured the DNA methylation of CpG sites in the 5′UTR region of *FGFR2* gene using Sequenom MassARRAY EpiTYPER approach (Sequenom, San Diego, CA, USA). This region located in chr10:123355182-123355644 (University of California, Santa Cruz, GRCh37/hg19 assembly). The amplicons of this region were generated by polymerase chain reaction using the sodium bisulphite-converted DNAs (Zymo Research, Orange, CA) as the templates. The primers for this region were designed using Epidesigner. The forward and backward primers were aggaagagagTATTGGGGTTTAGATTTTAGGAAGG and cagtaatacgactcactatagggagaaggctCAAAATACAAAAAAATTTTCTACCTCT. DNA methylation level was quantified by Honor Tech company (Beijing, China) using Sequenom MassARRAY platform [43] (San Diego, CA, USA). The methylation calls were performed by the EpiTYPER software v1.0 (Sequenom, San Diego, CA, USA), which generates quantitative results for each CpG site or an aggregate of multiple CpG sites. In total, our target region contains 16 CpG sites, and 15 out of the 16 sites could be measured independently except CpG 13, because its fragment molecular weight is too large and exceeds the detectable range. The molecular weight of CpG 3- and CpG 4-containing fragments overlap, which reduces the ability to resolve CpG 3 and CpG 4 methylation levels independently; thus, the DNA methylation of CpG 3.4 was estimated based on the average of the two CG-containing fragments. For details about this region, see Additional file 1: Tables S1 and S2. DNA methylation level (β) of each CpG site was calculated as the ratio of the intensity of methylated cytosines over the sum of the intensity of methylated and unmethylated cytosines. DNA methylation level ranges from 0 (unmethylated) to 1 (methylated).

### Quality control

To reduce batch effects, we tested half case and half control samples within each batch. To verify the internal replication, one random sample per batch was tested as a repeat. The difference of the methylation level between repeated samples was less than 5% across all CpG sites. To evaluate the efficiency of bisulphite conversion, one external unmethylated region of *INS* gene was tested in every batch as a conversion control. The bisulphite conversion efficiency of all samples was greater than 98%. In the samples’ quality control, we eliminated samples having a poor readout for each CpG. Then, we also removed the samples having DNA methylation level lower than 5% or higher than 95% for each CpG, due to the poor accuracy resulted from the detection limitation of Sequenom MassARRAY. In the following CpG quality control, we excluded the CpGs with less than 75% valid samples. Hence, our final analysis included CpG 2 of 130 samples (65 cases; 65 controls), CpG 3.4 of 160 samples (82 cases; 78 controls), CpG 5 of 138 samples (73 cases; 65 controls), CpG 7 of 133 samples (69 cases; 64 controls), and CpG 11 of 124 samples (69 cases; 55 controls). The flowchart of quality control is shown in Additional file 1: Figure S1.

### Statistical analyses

Continuous variables corresponding to normal distribution were described using mean and standard deviation. Categorical variables were described with proportions. Student’s *t* test and chi-squared test were used to compare the distributions of characteristics of mothers, placentas, and newborns.

We fitted a series of logistic regressions to explore the association of FT-LBW with DNA methylation and placental surface area, adjusting for maternal age, education, family monthly income, ETS exposure during pregnancy, gestational age, and newborn’s sex. To test the effect of prenatal ETS exposure on the findings, we conducted a sensitivity analysis in which the ETS did not include in the model as a covariate. Moreover, we performed another sensitivity analysis in which we excluded the mother-child pairs exposed to prenatal alcohol to test if the alcohol use influences the associations between DNA methylation levels of *FGFR2* and FT-LBW. Due to lack of the placental cell components’ measurements, we failed to adjust for cell type composition in our primary analysis. However, we generated an epigenome-wide dataset for a subset (*n* = 26) of the samples in our present study using Infinium Human Methylation 450K BeadChip (Illumina, San Diego, CA), following the standard manufacturer’s protocols. We displayed the process of quality control of the high-throughput data in Additional file 2, supplementary method. Surrogate variable analysis (SVA) approach [44] was used to identify the unmeasured sources of variation in the high-throughput DNA methylation data, which can confound the association between DNA methylation and FT-LBW. To test if our findings were driven by the unmeasured variations, in the subset samples, we compared the effect sizes of the individual CpG sites involved in our current study in the model adjusted for age, gender, and surrogate variables to the raw model adjusted for age and gender. The surrogated variables might capture the variations contributed by the cell type composition. Hence, we took the variations in the cell type composition into consideration. Additionally, we replicated the analysis and the comparison using the epigenome-wide 450k data. We performed the replication for only CpG 7 (cg25052156) that is the only CpG site measured independently by both EpiTYPER and Methylation 450K approaches.

We explored the association between DNA methylation and placental surface area using several multiple linear regression models, adjusting for all the confounders above. We also performed these models stratified by newborn’s sex, adjusting for the same confounders except newborn’s sex. To make the coefficients comparable across equations in mediation analysis, we used the standard score, which was calculated using *z* = (*x* − *μ*)/*σ*, of the placental surface area and DNA methylation in all the models. According to the method of Kenny and Sobel test, we considered the mediation being present when (a) the DNA methylation of any CpG sites at *FGFR2* gene was correlated with the placental surface area; (b) the DNA methylation of the CpG site(s) in (a) was/were correlated with FT-LBW without adjusting for the placental surface area; (c) the placental surface area was correlated with FT-LBW; and (d) the association between the DNA methylation of CpG site(s) in (a) and FT-LBW became weaker when the model was additionally adjusted for placental surface area. Using the method provided by VanderWeele [45], we calculated the direct and indirect effect of DNA methylation on FT-LBW; also, we estimated the proportion of the mediation effect of the placental surface area [46]. Mediation analysis was performed using “medflex” package in R 3.4.0, and the other analyses were conducted using SPSS 21.0 (SPSS Inc., Chicago, IL, USA). We applied Bonferroni correction and considered a *P* value less than 0.025 to be statistically significant.

## Results

### Population characteristics

Characteristics of all qualified subjects are presented in Table 1. On average, the mothers of FT-LBW newborns have lower education levels, lower family monthly income, and higher exposure to environmental tobacco smoke during pregnancy. None of the mothers have smoked ever or during pregnancy. As expected, the FT-LBW newborns had shorter body length and head circumference; also, their placentas were smaller and lighter.

**Table 1:** Maternal, placental, and fetal characteristics grouped by FT-LBW and FT-NBW

	FT-LBW (*N* = 86)	FT-NBW (*N* = 79)	*P* value
Mothers			
Age (year), mean (SD)	27.49 (5.88)	28.87 (4.28)	0.08
Pre-pregnancy BMI, mean (SD)	19.60 (2.66)	19.90 (2.20)	0.43
College or above, *N* (%)	27 (31.40)	40 (50.63)	0.03
Family income ≤ ¥3000/month, *N* (%)	57 (66.28)	34 (43.04)	0.003
Married, *N* (%)	84 (97.67)	76 (96.20)	0.67
Environmental tobacco smoke, *N* (%)	31 (36.05)	17 (21.52)	0.03
Primiparity, *N* (%)	64 (74.42)	56 (70.89)	0.51
Alcohol use, *N* (%)	5 (5.81)	2 (2.53)	0.45
Placenta			
Weight (g), mean (SD)	466.24 (54.58)	504.80 (56.07)	< 0.0001
Major axis length (cm), mean (SD)	18.60 (2.19)	19.86 (1.54)	< 0.0001
Minor axis length (cm), mean (SD)	17.24 (2.11)	18.90 (1.79)	< 0.0001
Area (cm^2^), mean (SD)	254.81 (60.72)	296.58 (48.07)	< 0.0001
Thickness (cm), mean (SD)	2.50 (1.86)	2.81 (3.42)	0.47
Newborns			
Female, *N* (%)	52 (60.47)	45 (56.96)	0.75
Gestational weeks (w), mean (SD)	38.02 (0.97)	38.04 (0.97)	0.92
Birth weight (g), mean (SD)	2317.27 (136.27)	3122.41 (349.60)	< 0.0001
Length (cm), mean (SD)	47.01 (1.48)	49.27 (1.42)	< 0.0001
Head circumference (cm), mean (SD)	31.36 (1.33)	33.48 (1.20)	< 0.0001

### Associations of DNA methylation of *FGFR2* in placenta with placental surface area and the risk of FT-LBW

**Fig. 1:**
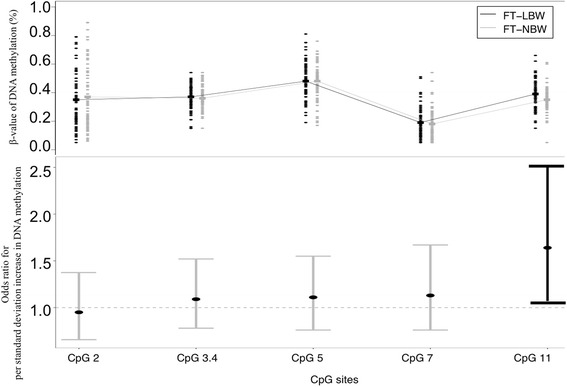
Differences of DNA methylation between groups. *β* value of DNA methylation distribution across CpGs grouped by FT-LBW and FT-NBW (upper panel). Comparison of the association between DNA methylation levels and FT-LBW across CpGs (lower panel)

Comparing to FT-NBW, DNA methylation levels of CpG 3.4, CpG 5, CpG 7, and CpG 11 were higher in FT-LBW, but CpG 2 methylation level was lower in FT-LBW (Fig. 1). The correlation coefficients (*r*) among CpG 2, CpG 3.4, CpG 5, CpG 7, and CpG 11 are shown in Additional file 1: Figure S2. We defined two blocks of dependent CpGs according to *r*. One block included CpG 2 and CpG 3.4 (*r* = 0.66), while the other block included CpG 5, CpG 7, and CpG 11 (*r* = 0.36 − 0.52). The correlation between the two blocks is negligible (*r* < 0.30) [47]. Hence, we performed Bonferroni correction for two independent tests. Comparing FT-LBW to FT-NBW, only the DNA methylation of CpG 11 at *FGFR2* was associated with an increased risk of FT-LBW (OR = 1.48; 95% CI = [1.01, 2.18]). When we further adjusted for the confounders, this association remained statistically significant with an effect size slightly greater (OR = 1.64; 95% CI = [1.07, 2.52]) (Table 2). Namely, per standard deviation increase in DNA methylation of CpG 11 in placenta was associated with 1.64-fold risk of FT-LBW. On average, FT-LBW babies had 4% higher methylation level of CpG 11 in placenta than FT-NBW babies (Table 2). In the stratification by newborn’s sex, this association was present and significant in girls (OR = 1.92; 95% CI = [1.07, 3.43]) but weaker and non-significant in boys (OR = 1.14; 95% CI = [0.52, 2.48]) (Table 2). In other words, per standard deviation increase in DNA methylation of CpG 11 in placenta was associated with 1.92-fold risk of FT-LBW in girls, but this association was not observed in boys. The mean methylation of CpG 11 was 6% higher in FT-LBW baby girls than in FT-NBW. Two sensitivity analyses demonstrated that the associations between CpG methylation of *FGFR2* and FT-LBW were robust in the model unadjusted for ETS (Additional file 1: Table S3) and in the subjects who had no prenatal alcohol exposure (Additional file 1: Table S4). We found an association between DNA methylation of CpG sites and placental surface area only at CpG 11 (*β* = − 0.18; 95% CI = [− 0.34, − 0.03], *p* = 0.02) (see the correlation scatter plots in Additional file 1: Figure S3). In the further sex-specific analysis, this association was found only in girls (*β* = − 0.21; 95% CI = [− 0.41, − 0.01]; *p* = 0.045) but not in boys (*β* = − 0.04; 95% CI = [− 0.30, 0.22]; *p* = 0.77). Placental surface area was significantly associated with FT-LBW (OR = 0.43; 95% CI, 0.28–0.68). This association was found significant in both girls (OR = 0.46; 95% CI = [0.26, 0.79]) and boys (OR = 0.38; 95% CI = [0.17, 0.87].

**Table 2:** Associations between the valid CpG methylation in placenta and risk of FT-LBW

*FGFR2* CpG sites	FT-LBW		FT-NBW		Odds ratio for per standard deviation increment in DNA methylation (95% CI)^a^	*P* value
	*N*	Mean (SD)	*N*	Mean (SD)		
All subjects						
CpG 2	65	0.35 (0.20)	65	0.37 (0.22)	0.95 (0.66, 1.37)	0.77
CpG 3.4	82	0.37 (0.08)	78	0.36 (0.08)	1.09 (0.78, 1.52)	0.62
CpG 5	73	0.48 (0.13)	65	0.48 (0.13)	1.09 (0.76, 1.55)	0.64
CpG 7	69	0.19 (0.13)	64	0.18 (0.11)	1.13 (0.76, 1.67)	0.54
CpG 11	69	0.39 (0.11)	55	0.35 (0.09)	1.64 (1.07, 2.52)	0.02
Girls						
CpG 2	40	0.33 (0.20)	35	0.39 (0.23)	0.85 (0.52, 1.37)	0.49
CpG 3.4	49	0.37 (0.08)	44	0.36 (0.07)	1.35 (0.84, 2.18)	0.49
CpG 5	44	0.49 (0.10)	39	0.47 (0.14)	1.19 (0.73, 1.95)	0.49
CpG 7	44	0.20 (0.15)	34	0.18 (0.11)	1.23 (0.72, 2.09)	0.45
CpG 11	43	0.40 (0.11)	32	0.34 (0.11)	1.92 (1.07, 3.43)	0.02
Boys						
CpG 2	25	0.37 (0.19)	30	0.33 (0.22)	1.32 (0.68, 2.57)	0.42
CpG 3.4	33	0.36 (0.09)	34	0.37 (0.08)	0.75 (0.43, 1.29)	0.29
CpG 5	29	0.47 (0.15)	26	0.49 (0.12)	0.84 (0.48, 1.47)	0.54
CpG 7	25	0.18 (0.10)	30	0.18 (0.12)	0.86 (0.42, 1.75)	0.67
CpG 11	26	0.38 (0.12)	23	0.36 (0.05)	1.14 (0.52, 2.48)	0.74

^a^In all subjects, the models were adjusted for maternal age, education, family monthly income, ETS exposure during pregnancy, gestational age and newborn’s sex. In girls and boys, the models were adjusted for all the covariates mentioned above, except the newborn’s sex

### Mediation analysis

**Fig. 2:**
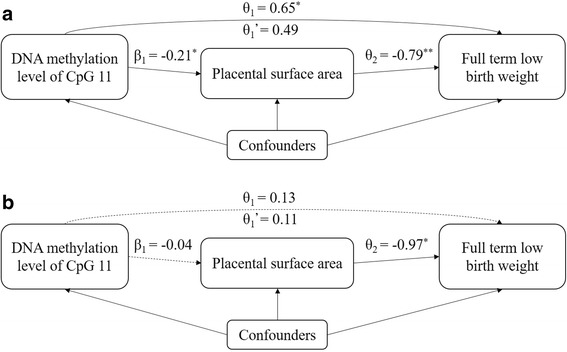
The mediation effects. Girls (**a**) and boys (**b**). *θ*_1_ is the estimated effect (ln OR) of CpG 11 methylation on FT-LBW adjusting for confounders. *θ*_1_’ is the estimated effect (ln OR) of CpG 11 methylation on FT-LBW additionally introducing placental surface area into model. *θ*_2_ is the estimated effect (ln OR) of placental surface area on FT-LBW adjusting for confounders. *β*_1_ is the coefficient of CpG 11 methylation on placental surface area. Confounders include maternal age, education, family monthly income, ETS exposure during pregnancy, and gestational age. *P value < 0.05; **P value < 0.01

**Fig. 3:**
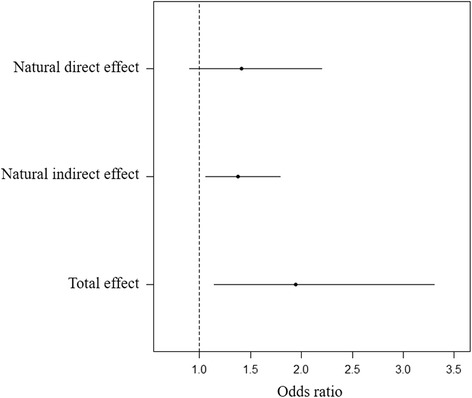
The decomposition of effect of the CpG 11 DNA methylation on FT-LBW

The association between the DNA methylation of CpG 11 and FT-LBW became weaker and non-significant when the model was additionally adjusted for the placental surface area in girls, compared to the model without adjustment of the placental surface area (Fig. 2a). However, in boys, the CpG 11 methylation was not linked to FT-LBW (Fig. 2b). Therefore, the mediation effect was present in girls and was not detected in boys, and the mediation analysis was only conducted in girls. The total effect was decomposed into a direct effect (OR = 1.41, 95% CI = [0.90, 2.22]) and an indirect effect (mediation effect) (OR = 1.38, 95% CI = [1.05, 1.80]) which attributed the effect of DNA methylation on FT-LBW through placental surface area (Fig. 3). The estimated mediation proportion was 48.38%.

## Discussion

In this study, higher methylation levels at of one novel CpG site (CpG 11, Chr10: 123355343) of *FGFR2* gene in the mother-side placental tissue were found in full-term low-birth compared to full-term normal-weight newborns. We also found that this association was more apparent in girls rather than in boys. Additionally, higher methylation level of CpG 11 was associated with lower placental surface area which was also confirmed to be associated with higher risk of FT-LBW. Moreover, the placental surface area played a role in mediating the association between CpG 11 DNA methylation and FT-LBW in girls.

Our findings demonstrated the association between increased placental *FGFR2* DNA methylation and FT-LBW. The link between DNA methylation of *FGFR* family and fetal growth has also been shown in previous studies. Haworth’s epigenome-wide study identified that DNA methylation of cg18566515 (not covered in our study) at *FGFR2* significantly increased in cord blood of newborns with birth weight centile greater than 85th compared to the medium birth weight centile (40th–60th) group. However, their study did not compare low birth weight centile (< 15th) group to the 40th–60th birth weight group [48]. In another epigenome-wide study, the DNA methylation level of cg15791248 at *FGFR1*, an important paralog of *FGFR2* [49], in cord blood was found negatively correlated with birth weight centile [50]. An animal study also demonstrated the role of *FGFR2* DNA methylation in growth rate [51]. Our study provides additional evidence in placental tissue of the link between *FGFR* family DNA methylation and fetal growth. However, the previous studies of the association between genome-wide DNA methylation in placenta and low birth weight did not found DNA methylation alternations of *FGFR2* [50, 52–55]. One possible reason for this inconsistency could lie in the coverage of the epigenome-wide experimental approaches. CpG 11 (Chr10: 123355343, UCSC GRCh37/hg19) found in our study was covered by neither Illumina HumanMethylation 27K nor 450K BeadChip; thus, it was impossible to detect CpG 11 in the previous epigenome-wide studies. Another possible reason might be the different definitions of the outcome. The previous studies were performed on birth weight (consisting of the premature and full term) or small for gestational age (SGA). In contrast, this study only focused on the full-term birth weight.

To explore the genetic variation’s influence on the association between DNA methylation of the target region of *FGFR2* and FT-LBW, we identified 156 methylation quantitative trait loci (mQTLs) in relation to CpG 3 (cg22633036) and CpG 7 (cg25052156) through performing a look-up in an online catalog of mQTLs (http://www.mqtldb.org) [56]. None of the 156 identified mQTLs was associated with birth weight according to an online genome-wide association study (GWAS) catalog (https://www.ebi.ac.uk/gwas) [57–60], which suggested that the associations of CpG 3 (cg22633036) and CpG 7 (cg25052156) DNA methylation with low birth weight might not be confounded by shared genetic variations. However, it is worth to mention that the mQTLs in the online catalog were identified in cord blood which may not reflect the specific mQTL status in placental tissue; moreover, we cannot identify the mQTLs related to the other CpG sites uncovered by the Illumine 450k methylation array. We further explored the DNA methylation level distribution of all valid CpGs and did not find a very frequent pattern that is an important characteristic of DNA methylation distribution influenced by genetic variations [61]. Therefore, the associations between DNA methylation modifications and FT-LBW were less likely driven by the genetic variations. More precise evaluation method and the process of the genetic variation’s effect should be developed and performed in the future study.

To explore the unmeasured variation’s influence on our findings, we estimated five significant surrogate variables in the subset samples of our present study using epigenome-wide methylation data and SVA approach and compared the effect size of the individual CpG sites in the model adjusted for age, gender, and surrogate variables to the raw model only adjusted for age and gender (Additional file 1: Table S5). We found that the effect sizes of CpG 2 and CpG 5 on FT-LBW in the adjusted models (adjusted for age, gender, and surrogate variables) changed as large as 151 and 29%, respectively, compared to those in model 0 (unadjusted for surrogate variables); however, the effect sizes of CpG 3.4, CpG 7, and CpG 11 on FT-LBW in the adjusted models changed less than 10% (2%, 10%, 6%), compared to those in model 0. It suggested that the associations of CpG 2 and CpG 5 with FT-LBW might be strongly driven by placental unmeasured variations while indicated a much weaker effect of the unmeasured variations on the associations of CpG 3.4, CpG 7, and CpG 11 with FT-LBW. This supplementary analysis made us more confident of our major positive finding (CpG 11). In the replication, we found that the effect size of cg25052156 (450k data) was much similar to the effect size of CpG 7 (EpiTYPER data) in both raw model 0 and adjusted model 3 and the directions of the effects were the same (Additional file 1: Table S5), which indicated consistency between two measurements. The effect size of cg25052156 on FT-LBW in model 3 increased 13% compared to that in model 0, which was also similar to the corresponding changes of CpG 7. One thing to note is that the evaluation of the unmeasured variation effect is based on very small samples that have different characteristic distributions from the entire population (Additional file 1: Table S6), which might not adequately reflect the real situation of our original study.

This study, for the first time, elucidated the association between the DNA methylation of *FGFR2* in placenta and placental characteristics. Existing biological evidence can help explain this association. The *FGFR2* gene expresses in placenta and upregulates trophoblast formation [31, 32]. If gene expression of *FGFR2* is inhibited, the embryo implantation and maternal-fetal interface formation will be interrupted [62], which can be reflected by reduced placental surface area [19]. Furthermore, FGFR2 affects placental angiogenesis and artery endothelial cell proliferation by inducing the FGF2 signal pathway [23–27]. Reduced trophoblast formation and peripheral villous vascularization lead to further intrauterine growth restriction [63]. It is consistent with these biological mechanisms that we observed the mediation effect of placental surface area on the association between *FGFR2* methylation and FT-LBW in girls. Although we did not measure the association between *FGFR2* methylation and gene expression due to lack of RNA samples, another study of thyroid cancer documented an inverse correlation between the DNA methylation of 5′UTR region at *FGFR2* and gene expression [64]. The region we measured in our study is also in the 5′UTR region, and this region locates in a predicted gene expression regulatory element (enhancer) region with a strong signal in Layered H3K27Ac [65]. This evidence highlights the probability of *FGFR2* DNA methylation as a regulation mechanism involved in *FGFR2* expression and underscores the importance of higher methylation of CpG 11 at *FGFR2* observed in our study relative to placental development and fetal growth.

It is well known that the placenta plays a key role in fetal growth. As a proxy for placental development, the placental surface area was found associated with fetal growth [20]. In our study, per one standard deviation increase in the placental surface area was associated with 0.45-fold lower risk of FT-LBW (OR = 0.45 for girls, OR = 0.37 for boys). In other studies, a 1-cm increase in placental axis was associated with 20.9–28.1 g higher birth weight [66]. Larger uteroplacental interface surface area in the first trimester associated with higher birth weight centile [67]. The surface area of the placenta at term was also highly and positively correlated with birth weight [68]. Our findings were in line with these studies.

The associations above between *FGFR2* DNA methylation in placenta, placental surface area, and FT-LBW suggested a mediation role of the placental surface area. In our study, we found that the association between *FGFR2* DNA methylation and FT-LBW became non-significant after being additionally adjusted for placental surface area, which proved the mediation effect. Placental surface area mediated 48.38% association between *FGFR2* DNA methylation and FT-LBW in girls in our study.

We found the associations of *FGFR2* methylation with the placental surface area and FT-LBW only in girls but not in boys. After stratifying for sex, we observed greater effect size of *FGFR2* methylation on FT-LBW in girls (OR = 1.92) compared to all (OR = 1.64). The sex-specific DNA methylation difference was also observed in other studies of birth weight. For example, in Kippler’s epigenome-wide study [69], *TSH7DA* (cg07846874) was found inversely associated with birth weight in girls but not in boys. On the contrary, cg19119945 was only observed in boys. The sex-specific DNA methylation changes with low birth weight were also detected in animal models [70, 71]. However, to our knowledge, none of the studies has characterized the sex-specific DNA methylation of *FGFR2* to compare with our findings. Our findings suggested that DNA methylation of CpG 11 at *FGFR2* has a sex-specific effect on placenta and fetal growth, but this might have been a question of low statistical power due to the limited sample size in boys.

Our study has several strengths. The outcome of this study focused specifically on full-term low birth weight which eliminated the effect of prematurity on birth weight and directly reflects the effect of intrauterine growth retardation. Secondly, we matched cases and controls for several characteristics which may influence both DNA methylation and birth weight. Thirdly, the mediation analysis method used in this study is specific for the binary outcome and continuous mediator, which allows analyzing the effect estimated with linear regression and logistic regression on the same scale.

Several limitations of the study should be addressed. Firstly, *FGFR2* gene expression regulates placental development mostly in the first trimester [28]; however, we collected placenta samples at delivery. Thus, we were unable to observe the pattern of placental *FGFR2* DNA methylation in the early and sensitive stage during pregnancy. Secondly, due to lack of the measures of placental cell type compositions and appropriate computational estimation methods for individual target DNA methylation, we failed to adjust the effects of cell type compositions on the results in this study. Thirdly, although the placental tissue samples were washed in physiological saline water to remove the blood tissue, the maternal DNA contamination effect may not be eliminated completely. Additionally, the placental surface area is not the exclusive proxy of uteroplacental interface development, but the density of vessel and villi are also important metrics. More placental morphology measurements should be performed in future studies. Furthermore, due to lack of RNA data, we were unable to estimate the association between DNA methylation of CpG 11 at *FGFR2* and *FGFR2* gene expression. The function examination of *FGFR2* methylation modification should be conducted in future studies.

## Conclusion

Our findings suggested that placental surface area mediates the association between DNA methylation of *FGFR2* in placenta and FT-LBW in a sex-specific manner. Our study supported the importance of placental epigenetic changes in placental development and fetal growth. The findings extend our understanding of the epigenetic mechanism of intrauterine growth.

## Additional files

Additional file 1: **Figure S1.** Quality control flowchart. **Figure S2.** The correlation of DNA methylation across all the valid CpG sites in valid samples. The correlation coefficients with the gray background are not statistically significant (*P* > 0.05). **Figure S3.** The scatter plots of placental surface area versus DNA methylation of CpG sites at *FGFR2*. The coefficient and *P* value were given by regression of placental surface area on DNA methylation using multiple linear regression model adjusted for maternal age, education, family monthly income, ETS exposure during pregnancy, gestational age and sex. (DOCX 294 kb)

Additional file 2: Includes the supplementary method. (DOCX 25 kb)
