# Supplementary material for: Placental surface area mediates the association between FGFR2 methylation in placenta and full-term low birth weight in girls
Source: Clin Epigenetics. 2018 Mar 22;10:39. doi: 10.1186/s13148-018-0472-5 (PMC5863829; doi:10.1186/s13148-018-0472-5)
Supplement: Supplementary file 1 — Figure S1. Quality control flowchart. Figure S2. The correlation of DNA methylation across all the valid CpG sites in valid samples. The correlation coefficients with the gray background are not statistically significant (P > 0.05). Figure S3. The scatter plots of placental surface area versus DNA methylation of CpG sites at FGFR2. The coefficient and P value were given by regression of placental surface area on DNA methylation using multiple linear regression model adjusted for maternal age, education, family monthly income, ETS exposure during pregnancy, gestational age and sex. (DOCX 294 kb) [file 13148_2018_472_MOESM1_ESM.docx]

**Additional materials**

**
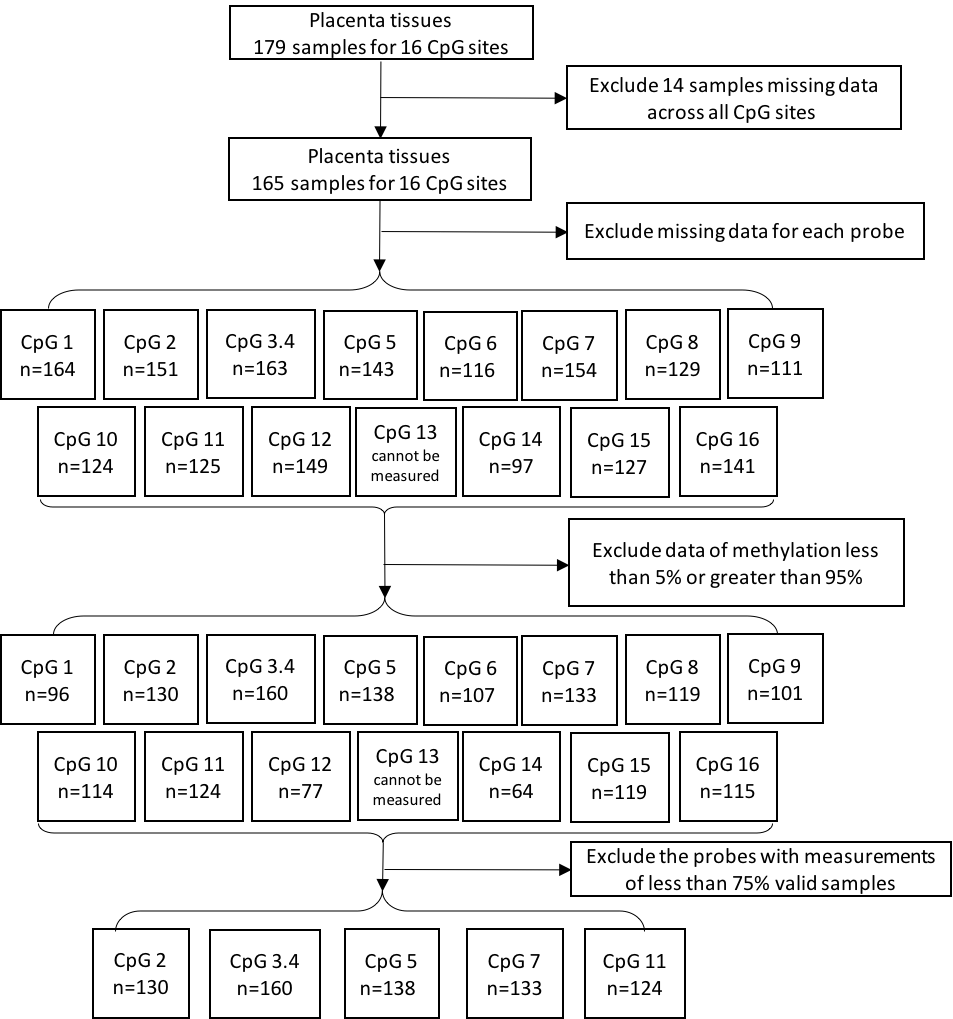
**

**Figure S1.** Quality control flow chart

**Table S1. PCR primers for *FGFR2* gene region.**

| Chromosomal Region^a^ | chr10:123355182 to 123355644 |
| --- | --- |
| Target sequence  (3^’^→5^‘^) | cagaatgcaggggaattttctacctctaacaC^[16]^GacttcaggaacaatcctaagtcctC^[15]^GcagaggtttcaccccaggctccttcccC^[14]^GgcctgctC^[13]^GcccacctgtccctgcccagaggagcctccttggctccaggcagagaaaggtggacagggC^[12]^GctcC^[11]^GctgaatctcaagcC^[10]^GttccatcatccC^[9]^GctagctggcactagaaatttgcatttcagccatttccaggtccaaaccccaC^[8]^GgcagccccctccccaaaactctggaaggC^[7]^GgagacctaaggC^[6]^GacaggtctgcacagttcctcacaccagtggacaaaagtatggtgC^[5]^GacctcctaggcaggataattaggtccccaggctC^[4]^GgtgttgggaatacaggcccagcatgcC^[3]^GctggC^[2]^GgccacaatccC^[1]^Ggcttcctgccttcaataccataatccttcctggagtctgagccccagtg |
| location | 5'UTR; N-shore |
| Primers (5^’^→3^’^) | Forward: aggaagagagTATTGGGGTTTAGATTTTAGGAAGG  Reverse: cagtaatacgactcactatagggagaaggctCAAAATACAAAAAAATTTTCTACCTCT |
| Amplicon length (bp) | 463 |

^a^ UCSC GRCh37/hg19

**Table S2. Genomic sites of methylation interrogated in the current study.**

| **Analytic Sites** | **Genomic location^a^** |
| --- | --- |
| CpG 1 | Chr10: 123355594 |
| CpG 2 | Chr10: 123355582 |
| CpG 3 | Chr10: 123355577 |
| CpG 4 | Chr10: 123355548 |
| CpG 5 | Chr10: 123355513 |
| CpG 6 | Chr10: 123355467 |
| CpG 7 | Chr10: 123355454 |
| CpG 8 | Chr10: 123355424 |
| CpG 9 | Chr10: 123355371 |
| CpG 10 | Chr10: 123355358 |
| CpG 11 | Chr10: 123355343 |
| CpG 12 | Chr10: 123355338 |
| CpG 13 | Chr10: 123355277 |
| CpG 14 | Chr10: 123355268 |
| CpG 15 | Chr10: 123355239 |
| CpG 16 | Chr10: 123355213 |

^a^ UCSC GRCh37/hg19

Grey shading CpG sites cannot be detected by Sequenom MassARRAY


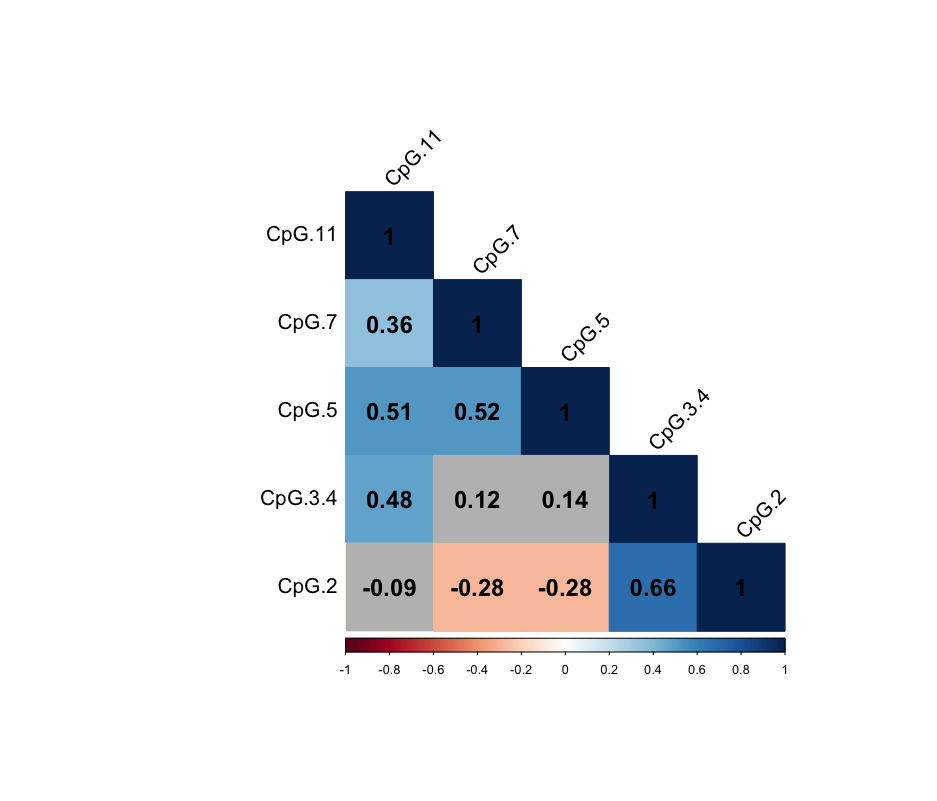


**Figure S2.** The correlation of DNA methylation across all the valid CpG sites invalid samples. The correlation coefficients with a grey background are not statistically significant (*P* > 0.05).


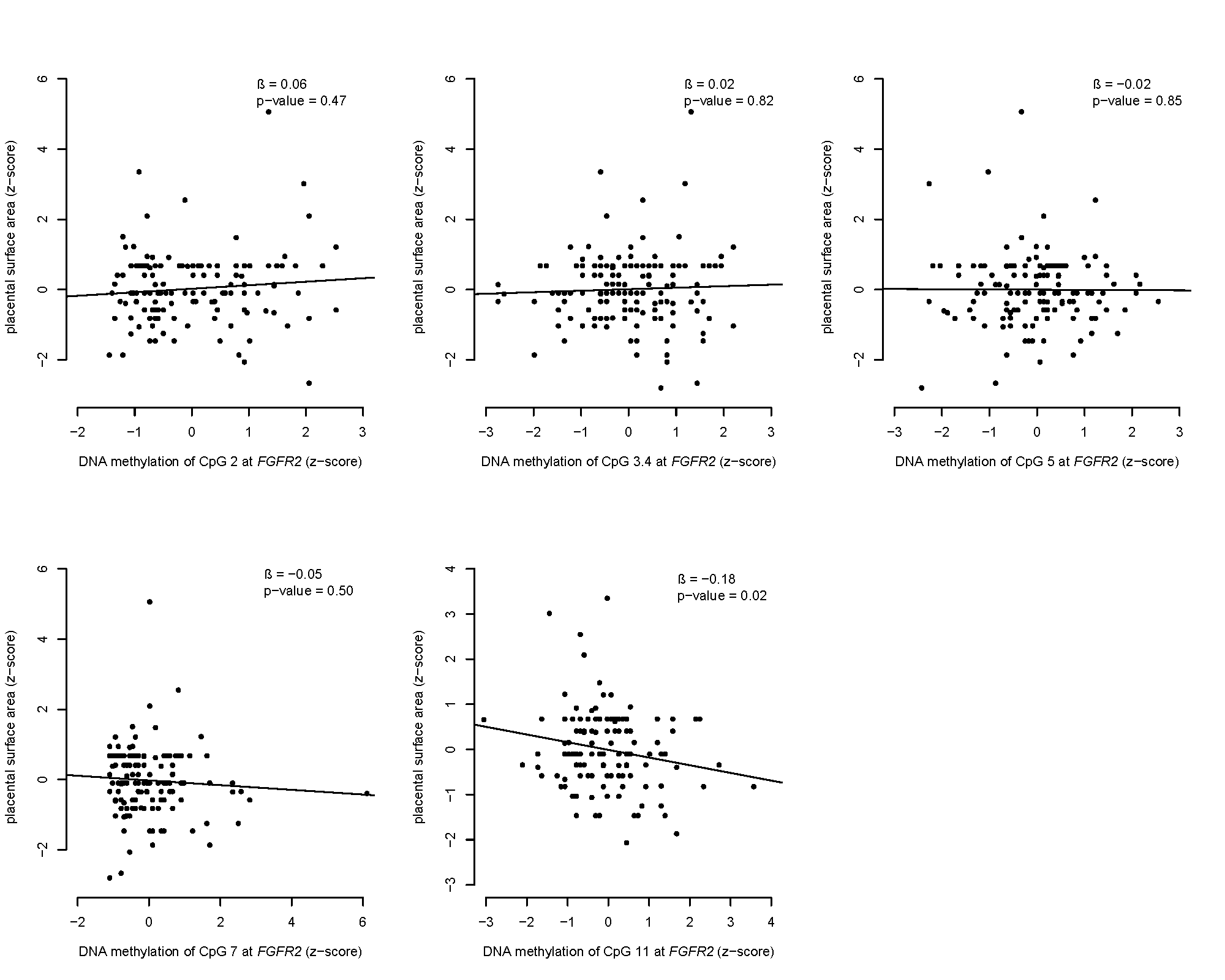


**Figure S3**. The scatter plots of placental surface area versus DNA methylation of CpG sites at *FGFR2*. The coefficient and *P*-value were given by regression of placental surface area on DNA methylation using multiple linear regression models adjusted for maternal age, education, family monthly income, ETS exposure during pregnancy, gestational age and sex.

**Table S3. Sensitivity analyses on the associations between the valid CpGs methylation of *FGFR2* in placenta and risk of FT-LBW.**

| **CpG sites** | N | | **Odds ratio for per standard deviation** **increment in DNA methylation (95% CI) ^a^** | ***P* - value** |
| --- | --- | --- | --- | --- |
|  | **FT-LBW** | **FT-NBW** |  |  |
| **All subjects** |  |  |  |  |
| CpG 2 | 65 | 65 | 0.93 (0.65, 1.35) | *0.71* |
| CpG 3.4 | 82 | 78 | 1.07 (0.77, 1.49) | *0.68* |
| CpG 5 | 73 | 65 | 1.11 (0.79, 1.58) | *0.54* |
| CpG 7 | 69 | 64 | 1.13 (0.76, 1.67) | *0.54* |
| CpG 11 | 69 | 55 | **1.61 (1.06, 2.45)** | ***0.02*** |
| **Girls** |  |  |  |  |
| CpG 2 | 40 | 35 | 0.76 (0.48, 1.21) | *0.25* |
| CpG 3.4 | 49 | 44 | 1.29 (0.81, 2.04) | *0.29* |
| CpG 5 | 44 | 39 | 1.27 (0.79, 2.03) | *0.33* |
| CpG 7 | 44 | 34 | 1.20 (0.73, 1.96) | *0.47* |
| CpG 11 | 43 | 32 | **1.90 (1.10, 3.31)** | ***0.02*** |
| **Boys** |  |  |  |  |
| CpG 2 | 25 | 30 | 1.27 (0.66, 2.45) | *0.47* |
| CpG 3.4 | 33 | 34 | 0.80 (0.47, 1.35) | *0.41* |
| CpG 5 | 29 | 26 | 0.83 (0.47, 1.46) | *0.52* |
| CpG 7 | 25 | 30 | 0.93 (0.47, 1.84) | *0.84* |
| CpG 11 | 26 | 23 | 1.31 (0.63, 2.71) | *0.48* |

^a^ In all subjects, the models were adjusted for maternal age, education, family monthly income, gestational age and newborn's sex. In girls and boys, the models were adjusted for all the covariates mentioned above, except the newborn's sex.

**Table S4. Sensitivity analyses on the associations between the valid CpGs methylation in placenta and risk of FT-LBW after excluding the subjects who had prenatal alcohol use.**

| ***FGFR2***  **CpG sites** | **FT-LBW** | | **FT-NBW** | | **Odds ratio for per standard deviation increment in DNA methylation (95% CI) ^a^** | ***P* - value** |
| --- | --- | --- | --- | --- | --- | --- |
|  | N | Mean (SD) | N | Mean (SD) |  |  |
| **All subjects** |  |  |  |  |  |  |
| CpG 2 | 60 | 0.35 (0.20) | 63 | 0.37 (0.23) | 1.01 (0.69, 1.47) | *0.98* |
| CpG 3.4 | 77 | 0.37 (0.08) | 76 | 0.37 (0.08) | 1.08 (0.77, 1.52) | *0.66* |
| CpG 5 | 69 | 0.49 (0.13) | 63 | 0.48 (0.14) | 1.07 (0.75, 1.54) | *0.70* |
| CpG 7 | 64 | 0.19 (0.14) | 62 | 0.18 (0.12) | 1.12 (0.75, 1.68) | *0.57* |
| CpG 11 | 65 | 0.39 (0.11) | 53 | 0.35 (0.09) | **1.76 (1.12, 2.77)** | ***0.01*** |
| **Girls** |  |  |  |  |  |  |
| CpG 2 | 37 | 0.35 (0.20) | 35 | 0.39 (0.23) | 0.94 (0.57, 1.54) | *0.79* |
| CpG 3.4 | 46 | 0.37 (0.08) | 44 | 0.36 (0.07) | 1.30 (0.81, 2.10) | *0.28* |
| CpG 5 | 42 | 0.49 (0.10) | 39 | 0.47 (0.14) | 1.17 (0.71, 1.92) | *0.54* |
| CpG 7 | 41 | 0.20 (0.15) | 34 | 0.18 (0.11) | 1.22 (0.72, 2.07) | *0.47* |
| CpG 11 | 41 | 0.40 (0.11) | 32 | 0.34 (0.11) | **2.03 (1.10, 3.76)** | ***0.02*** |
| **Boys** |  |  |  |  |  |  |
| CpG 2 | 23 | 0.37 (0.19) | 28 | 0.34 (0.22) | 1.51 (0.71, 3.24) | *0.29* |
| CpG 3.4 | 31 | 0.36 (0.09) | 32 | 0.37 (0.08) | 0.77 (0.44, 1.36) | *0.37* |
| CpG 5 | 27 | 0.48 (0.16) | 24 | 0.50 (0.12) | 0.82 (0.46, 1.47) | *0.51* |
| CpG 7 | 23 | 0.18 (0.10) | 28 | 0.19 (0.12) | 0.85 (0.41, 1.75) | 0.65 |
| CpG 11 | 24 | 0.39 (0.12) | 21 | 0.37 (0.05) | 1.34 (0.57, 3.15) | *0.50* |

^a^ In all subjects, the models were adjusted for maternal age, education, family monthly income, ETS exposure during pregnancy, gestational age, and newborn's sex. In girls and boys, the models were adjusted for all the covariates mentioned above, except the newborn's sex.

| *FGFR2*  CpG sites | N ^a^ | | Model 0 ^b^ | | Model 1 ^b^ | | Model 2 ^b^ | | Model 3 ^b^ | |
| --- | --- | --- | --- | --- | --- | --- | --- | --- | --- | --- |
|  | FT-LBW | FT-NBW | β-coefficient (95%CI) | *P-*value | β-coefficient (95%CI) | *P-*value | β-coefficient (95%CI) | *P-*value | β-coefficient  (95%CI) | *P-*value |
| CpG 2 | 10 | 12 | 5.61 (0.56, 56.09) | 0.14 | 5.40 (0.52, 56.54) | 0.16 | 5.86 (0.41, 84.32) | 0.19 | 14.06 (0.12, 1602.42) | 0.27 |
| CpG 3.4 | 11 | 12 | 3.01 (0.73, 12.45) | 0.13 | 3.07 (0.75, 12.63) | 0.12 | 3.21 (0.75, 13.73) | 0.12 | 3.08 (0.58, 16.41) | 0.19 |
| CpG 5 | 10 | 10 | 1.46 (0.60, 3.56) | 0.40 | 1.46 (0.60, 3.57) | 0.40 | 1.39 (0.53, 3.66) | 0.51 | 1.03 (0.20, 5.21) | 0.97 |
| CpG 7 | 12 | 11 | 1.11 (0.41, 3.01) | 0.84 | 0.98 (0.34, 2.79) | 0.97 | 1.07 (0.33, 3.47) | 0.91 | 1.22 (0.28, 5.35) | 0.79 |
| CpG 11 | 10 | 10 | 2.08 (0.58, 7.39) | 0.26 | 2.10 (0.59, 7.52) | 0.25 | 2.17 (0.56, 8.46) | 0.26 | 1.95 (0.25, 15.08) | 0.52 |
| cg25052156 (CpG 7) ^c^ | 14 | 12 | 1.10 (0.59, 2.00) | 0.81 | 1.15 (0.59, 2.22) | 0.68 | 1.15 (0.50, 2.64) | 0.75 | 1.25 (0.52, 3.77) | 0.51 |

**Table S5.** **Comparisons of the effect size across the CpG sites between the adjusted model (adjusted for age, gender and surrogate variables) and the raw model (adjusted for age and gender).**

a: The sample size for the individual CpG sites analyses was varied depending on the numbers of invalid samples excluded from analyses of each CpG site in the EpiTYPER data.

b: Model 0, adjusted for maternal age and child’s gender; Model 1, adjusted for maternal age, child’s gender and the first surrogate variable; Model 2, adjusted for maternal age, child’s gender and the first three surrogate variables; Model 3, adjusted for maternal age, child’s gender and all the five surrogate variables.

c: To replicate the association between DNA methylation of CpG 7 (EpiTYPER data) and FT-LBW using Methylation 450K data of cg25052156. CpG 7 in EpiTYPER approach is the site of cg25052156 in Methylation 450K approach.

**Table S6. Maternal, placental and fetal characteristics of the subset samples with the epigenome-wide DNA methylation data available.**

|  | **FT-LBW(N=14)** | **FT-NBW(N=12)** | ***P-value*** |
| --- | --- | --- | --- |
| **Mothers** |  |  |  |
| Age (y), mean (SD) | 26.14 (3.66) | 30.17 (4.26) | ***0.02*** |
| Pre-pregnancy BMI, mean (SD) | 18.99 (2.55) | 19.51 (3.09) | *0.64* |
| College or above, N (%) | 3 (21.40) | 6 (50.00) | *0.22* |
| Family income <=￥3000/month, N (%) | 9 (64.30) | 7 (58.30) | *1.00* |
| Married, N (%) | 13 (92.90) | 12 (100.00) | *1.00* |
| Environmental tobacco smoke, N (%) | 9 (64.30) | 7 (58.30) | *1.00* |
| Primiparity, N (%) | 11 (78.60) | 7 (58.30) | *0.40* |
| **Placenta** |  |  |  |
| Weight (g), mean (SD) | 438.25 (40.63) | 512.59 (74.45) | ***0.004*** |
| Major axis length (cm), mean (SD) | 19.00 (2.19) | 19.83 (1.90) | *0.41* |
| Minor axis length (cm), mean (SD) | 17.79 (2.01) | 18.83 (1.79) | *0.19* |
| Area (cm^2^) | 268.77 (68.87) | 295.77 (61.68) | *0.31* |
| Thickness (cm) | 2.07 (0.27) | 2.00 (0.00) | *0.37* |
| **Newborns** |  |  |  |
| Female | 6 (42.90) | 4 (33.30) | *0.70* |
| Gestational weeks, mean (SD) | 38.07 (0.92) | 38.42 (0.90) | *0.34* |
| Birth weight (g), mean (SD) | 2390.36 (87.17) | 3222.08 (353.70) | ***4.00 × 10^-6^*** |
| Length (cm), mean (SD) | 46.86 (1.03) | 49.25 (1.14) | ***8.00 × 10^-6^*** |
| HC (cm), mean (SD) | 30.93 (1.39) | 33.50 (1.31) | ***6.40 × 10^-6^*** |
