# Supplementary material for: Placental surface area mediates the association between FGFR2 methylation in placenta and full-term low birth weight in girls
Source: Clin Epigenetics. 2018 Mar 22;10:39. doi: 10.1186/s13148-018-0472-5 (PMC5863829; doi:10.1186/s13148-018-0472-5)
Supplement: Supplementary file 2 — Includes the supplementary method. (DOCX 25 kb) [file 13148_2018_472_MOESM2_ESM.docx]

**Additional file 2**

**Quality control of Infinium Human Methylation 450K data**

We inputted and preprocessed DNA methylation data using the *minfi* package of R [1]. In the sample quality control, we excluded samples that were mislabeled due to mismatch of genotype or sex. We also excluded low-quality samples with an individual call rate less than 98% and technical replicates (correlation coefficients of the replicates ranged from 0.98 to 1). In the probe quality control, we excluded SNP probes, probes on the X or Y chromosome, non-CpG probes, previously identified non-specific and cross-reactive probes [2], probes with poor detection (*P* > 0.05) for more than 1% samples, and SNP-associated probes (probe_SNP, CpG_SNP, SBE_SNP with minor-allele frequency >= 5%). In total, we included 334,980 CpGs (out of 485,577) in the analysis. We performed background correction and dye-bias equalization using the normal-exponential out-of-band (*noob*) correction method [3]. Furthermore, we applied the β-mixture quantile intra sample normalization procedure (BMIQ) to normalize the data [4]. Methylation level is represented by the average *β*-value = M/ (M+ U+ ε), where M and U represent the methylated and unmethylated CpG while ε usually equals 100. We used ComBat to correct batch effects in DNA methylation measurements [5].

**References:**

1. Aryee MJ, Jaffe AE, Corrada-Bravo H, Ladd-Acosta C, Feinberg AP, Hansen KD, et al. Minfi: a flexible and comprehensive Bioconductor package for the analysis of Infinium DNA methylation microarrays. Bioinformatics. 2014;30:1363–9.

2. Chen Y, Lemire M, Choufani S, Butcher DT, Grafodatskaya D, Zanke BW, et al. Discovery of cross-reactive probes and polymorphic CpGs in the Illumina Infinium HumanMethylation450 microarray. Epigenetics. 2013;8:203–9.

3. Triche TJ, Weisenberger DJ, Van Den Berg D, Laird PW, Siegmund KD. Low-level processing of Illumina Infinium DNA Methylation BeadArrays. Nucleic Acids Res. 2013;41:e90.

4. Teschendorff AE, Marabita F, Lechner M, Bartlett T, Tegner J, Gomez-Cabrero D, et al. A beta-mixture quantile normalization method for correcting probe design bias in Illumina Infinium 450 k DNA methylation data. Bioinformatics. 2013;29:189–96.

5. Johnson WE, Li C, Rabinovic A. Adjusting batch effects in microarray expression data using empirical Bayes methods. Biostatistics. 2007;8:118–27.
